# A comprehensive comparison of fecal microbiota in three ecological bird groups of raptors, waders, and waterfowl

**DOI:** 10.3389/fmicb.2022.919111

**Published:** 2022-08-08

**Authors:** Caiquan Zhao, Li Liu, Li Gao, Lige Bai

**Affiliations:** College of Biological Science and Technology, Baotou Teachers’ College, Baotou, China

**Keywords:** raptors, waders, waterfowl, fecal microbiota, co-occurrence network, metabolic pathways

## Abstract

Gut microbiota plays a vital role in maintaining the health and immunity of wild birds. However, less is known about the comparison of fecal microbiota between different ecological groups of wild birds, particularly in the Yellow River National Wetland in Baotou, China, an important transit point for birds migrating all over the East Asia-Australian and Central Asian flyways. In this study, we characterized the fecal microbiota and potential microbial function in nine bird species of raptors, waders, and waterfowl using 16S rRNA gene amplicon sequencing to reveal the microbiota differences and interaction patterns. The results indicated that there was no significant difference in α-diversity, but a significant difference in β-diversity between the three groups of birds. The fecal bacterial microbiota was dominated by Firmicutes, Proteobacteria, Actinobacteria, and Bacteroidetes in all groups of birds. Furthermore, we identified five bacterial genera that were significantly higher in raptors, five genera that were significantly higher in waders, and two genera that were more abundant in waterfowl. The bacterial co-occurrence network results revealed 15 and 26 key genera in raptors and waterfowls, respectively. The microbial network in waterfowl exhibited a stronger correlation pattern than that in raptors. PICRUSt2 predictions indicated that fecal bacterial function was significantly enriched in the antibiotic biosynthesis pathway in all three groups. Metabolic pathways related to cell motility (bacterial chemotaxis and flagellar assembly) were significantly more abundant in raptors than in waders, whereas waders were enriched in lipid metabolism (synthesis and degradation of ketone bodies and fatty acid biosynthesis). The fecal microbiota in waterfowl harbored more abundant vitamin B6 metabolism, RNA polymerase, and tyrosine and tryptophan biosynthesis. This comparative study revealed the microbial community structure, microbial co-occurrence patterns, and potential functions, providing a better understanding of the ecology and conservation of wild birds. Future studies may focus on unraveling metagenomic functions and dynamics along with the migration routine or different seasons by metagenomics or metatranscriptomics.

## Introduction

The coevolution of vertebrates and their gut microbiota can maintain homeostasis and improve the host dietary niches (Ley et al., 2008). An increasing amount of data from humans, cows, mice, chickens, insects, and fish has provided insight into the vital influence of gut microbes on host health, nutrition, immunity, and morphology (Muegge et al., 2011; Engel and Moran, 2013; Jing et al., 2021; Stressmann et al., 2021). Previous studies on the gut microbiota of bird species have focused on the effects of specific bacteria or bacterial pathogens, demonstrating the essential role of the gut microbiota (Benson et al., 2010). However, most of these studies have focused on only one or a few bird species. Few studies have compared the microbial community composition of bird species in different ecological groups, such as raptors, waders, and waterfowl, limiting our understanding of microbiota composition in multiple bird species.

Birds exhibit the most diverse range of ecological functions compared with other vertebrates and have a highly evolved lineage that is necessary for ecosystems (Zhan et al., 2017). Growing evidence has revealed that the dynamic gut microbial community of birds to adapt to their environment is affected by many factors such as host evolution, habitat environment, and human activity (Grond et al., 2019; Capunitan et al., 2020; San Juan et al., 2020). Additionally, the diet has been revealed as a major factor in the gut microbial composition in non-passerine birds (Xiao et al., 2021). A single host gene can have a significant effect on the diversity and structure of the gut microbiota (Spor et al., 2011). Furthermore, it has been shown that the gut microbiota of Darwin finches in the Galapagos Islands could be influenced by human activity (Knutie et al., 2019). Moreover, the habitat could also impact the gut microbiota of long-distance migratory swan geese (Wu et al., 2018). The gut microbiota of swan geese maintains a core group of species after long-distance migration, and it was found that host phylogeny is one of the main factors influencing the gut microbial community (Waite and Taylor, 2015). These studies provide a basic theoretical background for understanding gut microbiota and its function in different types of wild birds.

The significance of identifying the gut microbial community is that it provides a scientific basis for protecting endangered animals. Previous studies have confirmed that Proteobacteria is a diagnostic biomarker of reproductive dysfunction in crested ibises due to the abundance of Proteobacteria, and alpha-diversity indices were higher in sterile crested ibises than in healthy crested ibises (Ran et al., 2021). Similarly, the crested ibis chick growth rate was negatively associated with gut microbiota diversity and negatively correlated with the abundance of Dietziaceae, Halomonadaceae, Corynebacteriaceae, and Streptococci (Zhu et al., 2021). Furthermore, compared with the captive group of Alpine musk deer (*Moschus chrysogaster*), the Firmicutes-rich gut microbiota in the wild group enables individuals to maximize their energy intake from the cellulose, ensuring them to adapt to the wild environments (Sun et al., 2019). In addition, the fatal colibacillosis by MDR ESBL-producing *Escherichia coli* can threaten the critically endangered Brazilian merganser’s health, and in Orca whales, the potentially antibiotic-resistant pathogenic strains of *Escherichia coli* could contribute to the ongoing decline of this critically endangered population (Melendez et al., 2019; Fuentes-Castillo et al., 2021). Potential risks to human health will also be revealed by antibiotic-resistant bacteria in migratory birds because most wild birds are natural carriers of pathogenic bacteria (Cao et al., 2020). It is of significant importance to prevent the spread of infectious diseases in wild birds by studying the gut microbial composition (Zhao et al., 2018). The Yellow River National Wetland in Baotou is an important foraging location, which is a crossroad between the East Asia-Australian and Central Asian flyways, for migratory birds (Liu et al., 2019). A large number of different wild bird species inhabit this place. However, in recent years, the number of wild birds has declined owing to environmental changes and human activities. Therefore, it is urgently necessary to understand the gut microbiota structure and function in different wild birds in this region, providing an avenue for exploring the potential exchange of microbiota between different bird species and ecological groups.

In this study, we analyzed the fecal bacterial microbiota of three ecological bird groups (i.e., raptors, waders, and waterfowl), covering two endangered bird species, four national second-level protected species in China, and three common species at the Wildlife Conservation Center of Baotou (Inner Mongolia, China). Fecal microbial composition was identified by 16S rRNA gene amplicon sequencing, and microbiota function was predicted using PICRSUt2. The purpose of this study was to (i) reveal the fecal microbiota community structure, (ii) explore the microbial co-occurrence patterns, and (iii) compare the microbial potential function between raptors, waders, and waterfowl. This research enhances our understanding of the gut microbiota in different ecological groups of wild birds and provides novel insights into endangered bird conservation.

## Materials and methods

### Study objects and sample collection

All 38 fresh fecal samples were collected from three ecological groups of raptors (MQ), waders (SQ), and waterfowl (YQ), covering nine bird species, including endangered species of cinereous vulture (*Aegypius monachus*) and steppe eagle (*Aquila nipalensis*); the national second-level protected species in China, such as upland buzzard (*Buteo hemilasius*), common kestrel (*Falco tinnunculus*), demoiselle crane (*Anthropoides virgo*), and Eurasian Eagle-owl (*Bubo bubo*); common species of greylag goose (*Anser anser*), ruddy shelduck (*Tadorna ferruginea*), and black swan (*Cygnus atratus*) at the Wildlife Conservation Center of Baotou (Inner Mongolia, China) in August 2021 (Supplementary Table 1). The samples in this study were collected from wild birds (either wounded wings or wounded legs), which were rescued and protected at the Wildlife Conservation Center of Baotou. Fresh fecal samples were collected as soon as the birds arrived at the wildlife conservation center. The birds that met the following conditions were sampled: (i) fresh wounds, (ii) minor injuries, and (iii) no diseases. Groups of waders and waterfowl bird species had deposited stools. Only the middle layer of the fecal ball was collected from these different individuals. Freshly dropped raptor samples were collected by scraping or syringe suction from the surface (Becker et al., 2020). Samples were immediately transferred into 5-ml sterile tubes, placed on dry ice, and stored at −80°C until further processing.

### DNA extraction and sequencing

Genomic DNA was extracted from all the samples using the CTAB method (Honore-Bouakline et al., 2003). The extracted DNA was analyzed using a NANODROP LITE spectrophotometer (Thermo Scientific, United States) to evaluate DNA quantity and quality. We used the universal primers (341F [5′-CCTAYGGGRBGCASCAG-3′] and 806R [5′-GGACTACNNGGGTATCTAAT-3′]) to amplify the 16S rRNA gene V3–V4 region with 6 bp barcode unique to each sample. PCR was performed with 15 μl of Phusion High-Fidelity PCR Master Mix (New England Biolabs), 0.2 μM of forward and reverse primers, and 10 ng of template DNA. The PCR conditions were as follows: initial denaturation at 98°C for 1 min, followed by 30 cycles of denaturation at 98°C for 10 s, annealing at 50°C for 30 s, extension at 72°C for 30 s, and a final extension step at 72°C for 5 min. The PCR products were pooled and purified using the Qiagen Gel Extraction Kit (Qiagen, Germany). Illumina TruSeq^®^ DNA PCR-Free Sample Preparation Kit (Illumina, United States) was used to produce sequencing libraries according to the manufacturer’s recommended protocol. After the detection of library quality, samples were sequenced using the Illumina NovaSeq platform with 250 bp paired-ended running mode. The sequencing service was provided by Novogene Co., Ltd., Tianjin, China. Raw reads were deposited and are available through the Sequence Read Archive under accession number SRR18553438.

### Sequencing data processing

All raw paired-end sequences were imported to the QIIME2 pipeline (version 2020.8.0) (Bolyen et al., 2019). Primers were removed using the Cutadapt plugin by “qiime cutadapt trim-paired” (–p-minimum-length 200). The DADA2 plugin (“qiime dada2 denoise-paired”) was used to generate denoised feature sequences (amplicon sequence variants, ASVs) and feature tables (-p-trim-left-f 0 –p-trim-left-r 0 –p-trunc-len-f 235 –p-trunc-len-r 220) (Callahan et al., 2016). Feature sequences with frequency ≤ 4 were discarded using “qiime feature-table filter-features –p-min-frequency 4.” Reference sequences were extracted from the SILVA database (release 132) using specific primers for the 16S V3-V4 region using “qiime feature-classifier extract-reads –p-min-length 200 –p-max-length 500” (Quast et al., 2013). The Naive Bayes classifier was trained for taxonomic annotation by the command line of “qiime feature-classifier fit-classifier-naive-bayes.” The ASVs assigned to mitochondria and chloroplast were excluded from the feature table (“qiime taxa filter-table –p-exclude mitochondria, chloroplast”) and feature sequences (“qiime taxa filter-seqs –p-exclude mitochondria, chloroplast”). We use the PICRUSt2 (Phylogenetic Investigation of Communities by Reconstruction of Unobserved States) software^1^ to predict the functional abundance of microbiota (Douglas et al., 2020).

### Statistical analysis

Alpha-diversity indices of microbiota (Chao1, Simpson, and Shannon) were calculated using the command line of “qiime diversity alpha.” These indices between groups were compared using the non-parametric Wilcoxon rank-sum test. Principle coordinate analysis (PCoA) plot was generated based on the Bray–Curtis distance. Permutational analysis of variance (PERMANOVA) was applied to test group differences based on Bray–Curtis distance matrix using vegan package (Oksanen et al., 2007). LEfSe (Linear Discriminant Analysis Effect Size) was used to test the difference in taxa abundance (LDA score ≥ 4.0 and *p* < 0.05) and functional abundance (LDA score ≥ 3.0 and *p* < 0.05) (Segata et al., 2011). The interaction network was constructed using a genus present in ≥60% of samples in MQ and YQ, respectively. Briefly, (i) we calculated the correlation coefficient using SparCC algorithm (Watts et al., 2019), (ii) the statistical significance of correlations was calculated from 1,000 bootstrap iterations, (iii) network (*| SparCC|* > 0.5 and adjusted *p* < 0.05) property calculation and module detection were employed using the igraph package, and (iv) Network visualization was performed using the Cystoscope 3.7.1 software (Shannon et al., 2003).

## Results

### Diversity of fecal microbiota in raptors, waders, and waterfowl

**FIGURE 1:**
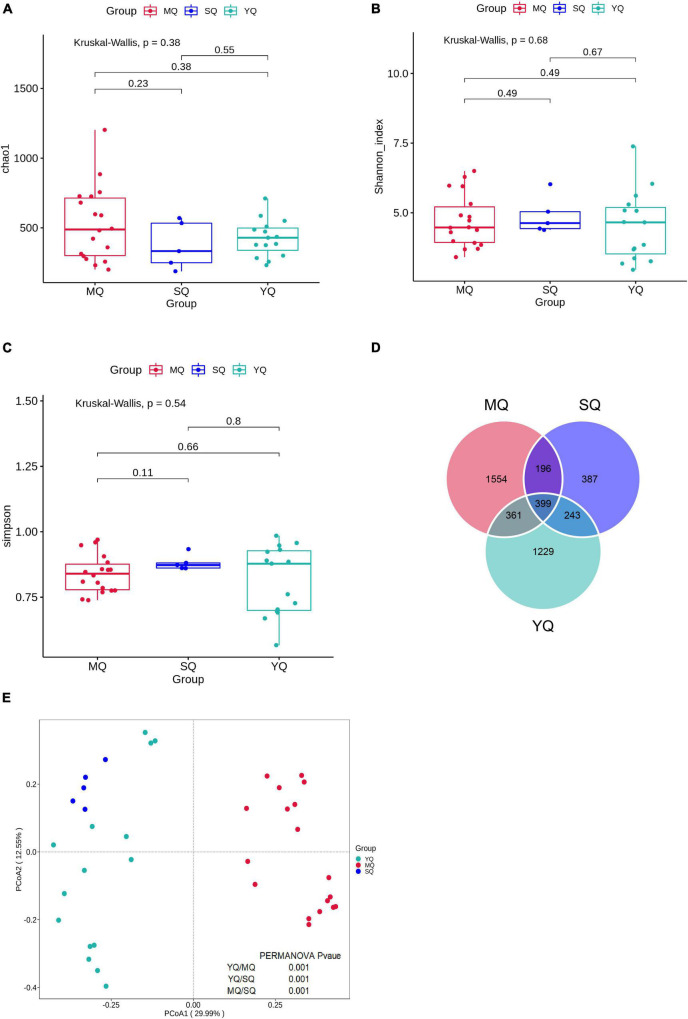
Alpha and beta diversity of fecal microbiota among three ecological groups of birds (MQ, SQ, and YQ). Alpha diversity was characterized by Chao1 index **(A)**, Shannon index **(B)**, and Simpson indices **(C)**. Solid horizontal line within a box represents the median, the dots indicate the observed value, the box margins are the interquartile range (50% of the observations), and whisker lines extend for 1.5 times the interquartile range. There was no significant difference in Chao1 index, Shannon index, and Simpson indices (*p* > 0.05). Venn diagram of ASVs overlapping across MQ, SQ, and YQ based on ASV presence and absence **(D)**. Principal coordinate analysis of fecal bacterial communities from the three groups of birds **(E)**. MQ, raptors; SQ, waders; YQ, waterfowl.

After quality control, a total of 2,139,722 effective sequences were obtained from all samples, with an average of 76,387 ± 4,908 (mean ± SD) in each sample (Supplementary Table 2). These sequences were denoised using DADA2 to obtain 6,807 amplicon sequence variants (ASVs). The rarefaction curve revealed an adequate sequence depth for covering the fecal bacterial community in birds based on the number of observed features and the Shannon index (Supplementary Figure 1). To determine the variations in fecal microbiota in MQ, SQ, and YQ, we characterized alpha diversity using the Chao1, Shannon, and Simpson indices. The results showed no significant differences among the three groups of birds (Figures 1A–C). The total number of ASVs in the MQ and YQ was significantly higher than that in the SQ (Supplementary Figure 1A). Although there was no significant difference in alpha diversity among the three groups of birds, Venn analysis indicated that MQ and YQ possessed 1,554 and 1,229 unique ASVs, respectively, more than that of SQ (387). Only 399 common ASVs were found in all three bird groups (Figure 1D). Principal coordinates analysis (PCoA) showed group-based separation of microbial communities based on Bray–Curtis dissimilarity (Figure 1E). PERMANOVA also verified that each ecological group had a significantly different microbial community structure (YQ vs. MQ: *R*^2^ = 0.288, *p* < 0.05; YQ vs. SQ: *R*^2^ = 0.200, *p* < 0.05; MQ vs. SQ: *R*^2^ = 0.319, *p* < 0.05). Moreover, there was a significant difference between the nine bird species (Supplementary Figure 2 and Supplementary Table 3).

### The fecal bacterial community in raptors, waterfowl, and waders

**FIGURE 2:**
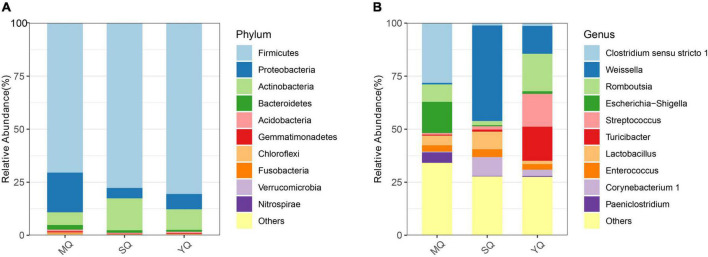
Bar chart of relative abundance. The relative abundance (%) of the top 10 abundant bacteria phyla **(A)** and genera **(B)** among the three ecological bird groups. The dominant phyla in three groups of birds consisted of Firmicutes, Proteobacteria, Actinobacteria, and Bacteroidetes. Others, bacteria taxa with ≤1% abundance. MQ, raptors; SQ, waders; YQ, waterfowl.

All the ASVs were assigned to 40 phyla, 103 classes, 235 orders, 387 families, and 815 genera (including unclassified entries). We analyzed the bacterial composition of the fecal samples at the phylum and genus levels. The dominant phyla in the three ecological groups of birds were Firmicutes (70.49% in MQ, 77.71% in SQ, 80.55% in YQ), Proteobacteria (18.69%, 4.87%, 7.21%), Actinobacteria (6.02%, 15.09%, 9.71%), and Bacteroidetes (2.15%, 1.15%, 0.86%) (Figure 2A). As is shown in Figure 2B (Supplementary Table 4), the genus of *Clostridium sensu stricto 1* occupied 28.16% in MQ, but only about 1.12% and 1.34% in the other two groups of SQ and YQ, respectively. We also observed increased genera in MQ but decreased genera in the other two groups, namely, *Escherichia-Shigella* and *Paeniclostridium*. The top four genera in the SQ were *Weissella* (45.01%), *Corynebacterium 1* (8.91%), *Lactobacillus* (8.33%), and *Staphylococcus* (5.79%) (Supplementary Table 4). YQ was dominated by *Romboutsia* (17.68%), *Turicibacter* (16.05%), *Streptococcus* (15.50%), and *Weissella* (13.08%).

### Bacterial co-occurrence patterns in raptors and waterfowl

**FIGURE 3:**
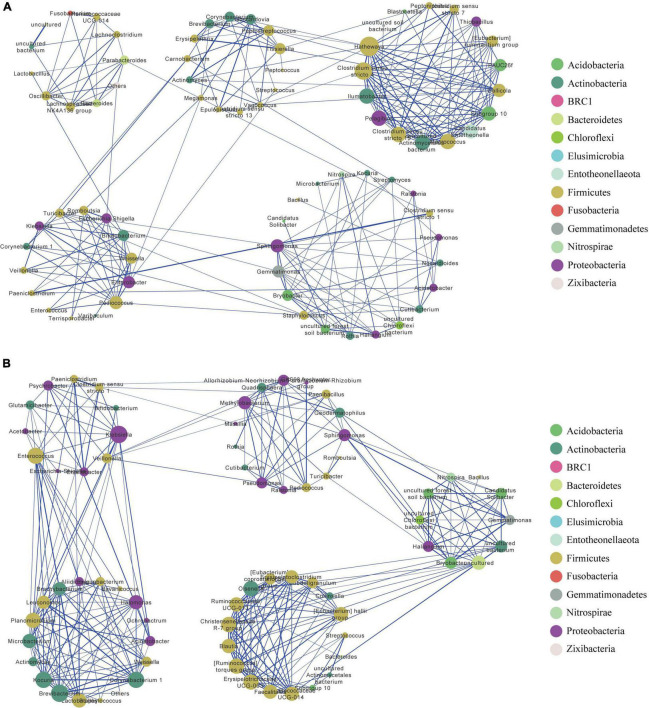
Bacterial co-occurrence network in MQ **(A)** and YQ **(B)** based on positive correlation analysis at the genus level. Nodes correspond to genus and edges to the correlation. Node size is proportional to the degree number. Node color represents the associated phylum for each genus. Edge width displays the strength of correlation. The blue edge indicates a positive correlation. Each large circle (e.g., MQ-M1 or YQ-M1) represents a module detected by Louvain method. MQ, raptors; SQ, waders; YQ, waterfowl.

The resulting network in MQ consisted of 78 nodes and 250 edges, with an average degree of 6.41. The clustering coefficient was 0.52, and network modularity was 0.66. We detected five modules in the MQ group, in which MQ-M5 was a highly connected sub-network (Figure 3A). The MQ-M5 module, mainly assigned to Firmicutes, had the highest degree (≥10) of nodes, defined as key nodes in this study, including the uncommonly seen genera *Hathewaya* (0.77%), *Clostridium sensu stricto 4* (0.26%), *Clostridium sensu stricto 15* (0.80%), *Macrococcus* (0.19%), *uncultured Actinomycetales bacterium* (0.15%), *Pelagibius* (0.05%), and *Ilumatobacter* (0.04%). However, the high relative abundances of genera, such as *Clostridium sensu stricto 1* (28.2%), *Escherichia-Shigella* (14.7%), *Romboutsia* (8.22%), *Paeniclostridium* (4.90%), and *Lactobacillus* (4.53%), were low-degree nodes. Most of the key nodes had a substantial proportion of intra-module degree (>60%) and a small proportion of intra-module degree (Supplementary Table 5), indicating a tight intra-connection and sparse inter-connection in the MQ microbiota interaction network. The network in YQ contained 71 nodes and 286 edges with an average degree of 8.06, a clustering coefficient of 0.60, and a modularity of 0.61 (Figure 3B). Both MQ and YQ networks exhibited clustered topologies and modular structures. There were 26 key nodes in the YQ network (Supplementary Table 6). In YQ-M1 (mostly assigned to Proteobacteria and Firmicutes), the nodes of *Klebsiella* (0.80%) and *Enterococcus* (2.68%) had a large degree number of 15 and 14, respectively. *Enterococcus* was particularly associated with YQ-M3, in which most nodes belonged to Proteobacteria, Firmicutes, and Actinobacteria. YQ-M3 contained nine key nodes, among which *Brevibacterium* (relative abundance of 0.503%) had the largest degree number of 15, and *Corynebacterium 1* was observed for the highest relative abundance of 2.99%. YQ-M4, which was mainly mapped to Firmicutes, was also a large module with nine key nodes. Interestingly, the modules in the bacterial network of YQ were stronger than those in MQ based on the proportion of inter-module degree (Supplementary Table 6), such as YQ-M1, YQ-M3, YQ-M5, YQ-M2, YQ-M4, and YQ-M5. Detailed statistical information and negative associations between the microbial co-occurrence networks in raptors and waterfowl are shown in Supplementary Figure 3 and Supplementary Tables 7, 8.

### Differences in bacterial taxa between raptors, waders, and waterfowl

**FIGURE 4:**
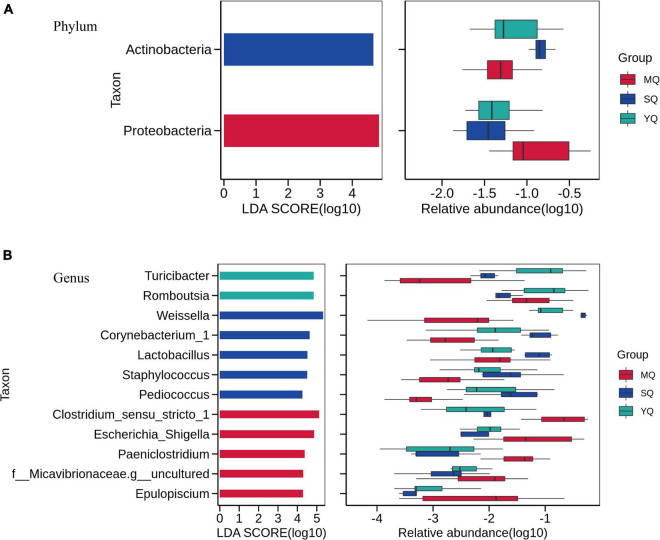
Linear discriminant analysis effect size (LEfSe) analysis. The linear discriminant analysis identified significantly different taxon between MQ, SQ, and YQ at the phylum **(A)** and genus **(B)** levels with a threshold of LDA score ≥ 4.0 and *p* < 0.05. Left, logarithm score of LDA analysis for each taxon. Right, relative abundance of different taxon; MQ, raptors; SQ, waders; YQ, waterfowl; LDA, linear discriminant analysis; LEfSe, LDA Effect Size.

Linear discriminant analysis effect size was performed to reveal differences in the bacterial microbiota between MQ, SQ, and YQ. The results showed that the biomarkers at the phylum level were Actinobacteria in SQ and Proteobacteria in MQ (LDA score ≥ 4, *p* < 0.05) (Figure 4A). At the genus level, we observed biomarkers for 12 taxa, namely, *Turicibacter*, *Romboutsia*, *Weissella*, *Corynebacterium_1*, *Lactobacillus*, *Staphylococcus*, *Pediococcus*, *Clostridium_sensu_stricto_1*, *Escherichia_Shigella*, *Paeniclostridium*, *Micavibrionaceae.g_uncultured*, and *Epulopiscium* (LDA ≥ 4.0, *p* < 0.05) (Supplementary Table 9). Among these 12 taxa, five genera were significantly higher in MQ, five were higher in SQ, and two were higher in YQ (Figure 4B). Moreover, there were five, six, nine, and nine significantly different taxa at the class, order, family, and species levels, respectively (Supplementary Figure 4).

### Potential function of the fecal bacterial microbiota in raptors, waders, and waterfowl

**FIGURE 5:**
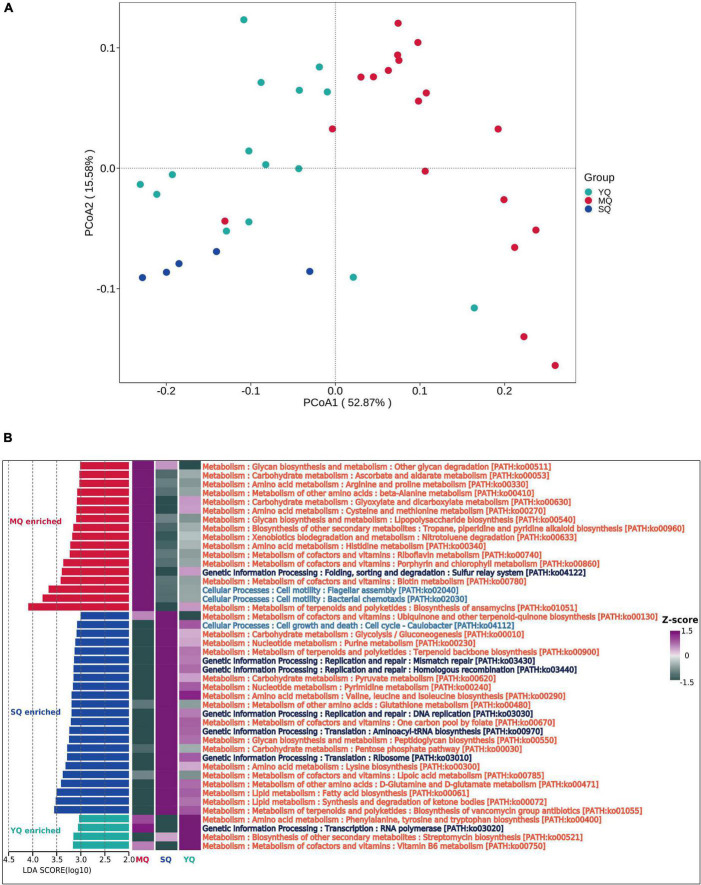
Microbial functional difference analysis between groups. **(A)** KOs principal coordinate analysis (PCoA) based on Bray–Curtis distance of predicted KO abundance. **(B)** Difference in the KEGG pathway identified by LEfSe (LDA score ≥ 3.0 and *p* < 0.05) between MQ, SQ, and YQ. Heat maps of differential pathways enrichment analysis based on KEGG. MQ, raptors; SQ, waders; YQ, waterfowl.

To explore the functional profiling of the bacterial microbiota in different ecological bird groups, PIPCRUSt2 was used based on a 16S rRNA gene marker. PCoA results indicated significant clustering by diverse groups of birds based on the predicted KEGG ortholog (KOs) abundance (Figure 5A, YQ vs. MQ: *R*^2^ = 0.271, *p* < 0.01; YQ vs. SQ: *R*^2^ = 0.166, *p* < 0.01; MQ vs. SQ: *R*^2^ = 0.398, *p* < 0.01), which was consistent with the microbial community structure (Figure 1E). To determine the difference in metabolic potential in the fecal microbiota, the KEGG pathway was analyzed. LEfSe results (LDA score ≥ 3.0, *p* < 0.05) showed that the enriched functional classes in MQ mainly included biosynthesis of ansamycins (ko01051), bacterial chemotaxis (ko02030), and flagellar assembly (ko02040). The YQ group was dominated by vitamin B6 metabolism (ko00750); streptomycin biosynthesis (ko00521); RNA polymerase (ko03020); and phenylalanine, tyrosine, and tryptophan biosynthesis (ko00400). Functional pathways involved in the biosynthesis of vancomycin group antibiotics (ko01055), synthesis and degradation of ketone bodies (ko00072), and fatty acid biosynthesis (ko00061) were upregulated in the SQ group. In summary, there was a significant difference in fecal microbiota function among the three groups of birds (Figure 5B).

## Discussion

Gut microbiota exerts a major influence on food digestion, metabolism regulation, and immune protection in wild animals (Nicholson et al., 2012; Valdes et al., 2018). Both host species and diet have been suggested to be potential drivers modulating the gut microbial composition of wild animals (Xiao et al., 2021). The Yellow River National Wetland in Baotou, China, is an indispensable feeding station for migratory birds between the East Asian-Australian and Central Asian flyways (Liu et al., 2022). Thus, we selected wild birds to study the differences in the composition of the gut microbiota.

In this study, we compared the fecal microbiota composition of raptors, waders, and waterfowls. Although live in the Yellow River National Wetland in Baotou, these ecological birds have different habitat preferences. Waders have physical and behavioral adaptations for living near water. They live in waterflow areas, are strong swimmers, and spend much of their time on ponds, lakes, or rivers. Raptors can be found in tundra, desert, forests, and grasslands. Different living habit makes birds have different dietary preferences. It was observed that there was an insignificant difference in alpha diversity, but a significant difference in beta diversity, showing consistent species richness and evenness within fecal microbial communities in the three groups of birds, but a considerable divergence in microbial profiles between these groups. Our data showed that Firmicutes, Proteobacteria, Actinobacteria, and Bacteroidetes were the dominant phyla, which is supported by several previous studies (Xenoulis et al., 2010; Waite et al., 2012; Dewar et al., 2013, 2014; Wang et al., 2016a,b; Zhao et al., 2017). Firmicutes can provide an energy source to hosts through the metabolism of polysaccharides, sugars, carbohydrates, and fatty acids (Flint et al., 2008; Tap et al., 2009). Most Actinobacteria are widely distributed in water and soil. These bacteria are easily transmitted to birds, suggesting that the bacterial community structure of birds is influenced by external bacterial transmission (Mikaelyan et al., 2015). Actinobacteria, which dominant in waders, are closely related to host fiber intake (Lee et al., 2015). This is consistent with the fact that the waders and waterfowl dietary preference of plants and grains. Carnivorous raptors are often regarded as pathogen vectors (Zhou et al., 2020). Consistent with previous studies, our research showed that the dominant flora of the gut microbiota of raptors were Proteobacteria, which have higher relative abundances than waders and waterfowl. It has been reported that Proteobacteria harbors the most microbial pathogens, such as *Escherichia*, *Shigella*, and *Paeniclostridium*, which were also detected in this study in raptors and could cause enteric disease in animals or diarrhea in humans (Kotloff et al., 2013; Nyaoke et al., 2020). *Epulopiscium* spp., which are dominant in the gut of raptors, are a group of large gram-positive bacteria that have a symbiotic relationship with their hosts and generally inhabit the gut of fish (Angert, 2021). *Clostridium sensu stricto 1*, also a significantly higher proportion of genera in raptors, is mostly a strictly anaerobic, fermenting bacteria. *Clostridium perfringens*, a member of *Clostridium sensu stricto 1*, was more abundant than any other member in this study (Supplementary Figure 4). *C. perfringens* is a rapidly growing opportunistic pathogen associated with intestinal diseases in humans and animals that secretes more than 20 virulent toxins (Kiu and Hall, 2018). In the fecal microbiota of waders and waterfowl, most genera, including *Turicibacter*, *Romboutsia*, *Weissella*, and *Lactobacillus*, were demonstrated as commensal bacteria, which were mainly driven by a diet with fiber-rich plant and grains, to maintain host health status and provide a host energy source by inhibiting inflammatory reactions, antimicrobial activity, or specific metabolic capability (Liu et al., 2014; Zhong et al., 2015; Milani et al., 2016; Ricaboni et al., 2016; Hernandez-Oaxaca et al., 2021). Besides, the usual habitat, migration paths, and host evolutionary also impact on the gut bacterial composition of birds (Loo et al., 2019; Lee et al., 2020; Skeen et al., 2021). Nevertheless, further exploration is still necessary to ensure taxonomic accuracy due to the low resolution of amplicon sequencing.

Correlation-based network analysis offers new insights into microbial community structure and its co-occurrence patterns, showing ecological interactions (Yeung et al., 2021). Modularity is an important indicator of ecosystem stability and resilience (Sharaf et al., 2019). The modularity of the microbial network in waders was greater than that in raptors, which may be the cause of the number of pathogens in the raptor gut. Node degree (or connectivity) is simply a measurement of the activity or importance it represents in a network (Wasserman and Faust, 1994). *Hathewaya limosa* (*Clostridium limosum*), which is usually found in animals and birds, is a pathogen that causes inflammatory diseases (Cato et al., 1970; Bistrom et al., 2016). In this study, there was a large degree of both positive and negative interaction networks in raptors (Supplementary Tables 5, 7). *Clostridium sensu stricto 4* (also called *Clostridium cluster IV* or *Clostridium leptum* group) can produce short-chain fatty acids, and its decrease is associated with the expansion of gut pathogens (Livanos et al., 2018). We observed that *Clostridium sensu stricto 4* had several negative connections with potential pathogens in raptor feces, such as *Escherichia-Shigella*, *Paeniclostridium*, *Staphylococcus*, *Klebsiella*, and *Enterobacter* (Supplementary Figure 3A). This may be due to the balance between beneficial microbes and pathogens, which maintains the stability of gut microecology. Additionally, there were also some pathogens, such as *Klebsiella* and *Enterococcus*, dominating the connectivity in the positive correlation network of waders (Figure 3B), which might be due to polluted water or soil. We speculated that these pathogens had an impact on the microbial co-occurrence patterns in the ecological bird groups. The clustering coefficient is typically used to describe the hierarchical properties of a network, representing how nodes are embedded in their neighborhood (Deng et al., 2012). This index in the microbial network of raptors was higher than that in waders, indicating a higher co-association among microbes in the gut of waders.

Although many microbial pathogens inhabit the guts of wild birds, the birds themselves are not affected by infection. It might be that the antimicrobial agents produced by the host or some commensal bacteria maintain a biological balance. This hypothesis was verified by the predicted microbial function in this study, in which we found that the pathways of antibiotic biosynthesis were enriched in the fecal microbiota of all ecological bird groups, such as ansamycins in raptors, streptomycin in waterfowl, and vancomycin in waders. Moreover, cell motility-related pathways, mainly flagellar assembly and bacterial chemotaxis, were overrepresented in raptor fecal microbiota, which may be driven by a high-fat diet (Hildebrandt et al., 2009). Ketone bodies are usually produced from the breakdown of fatty acids by ketogenesis in the liver during low food intake, carbohydrate-restrictive diets, or host starvation (Rui, 2014). This could offer an energy source for the host brain (Hasselbalch et al., 1994). The gut microbiota driven by ketone bodies also plays a key role in recovering the host immune imbalance (Cabrera-Mulero et al., 2019). Vitamin B6, an essential nutrient for animal hosts, is vital for regulating amino acid balance. However, the animals cannot produce it themselves, and it mainly comes from intestinal bacteria and diet. Inflammatory diseases occur due to vitamin B6 deficiency (Rall and Meydani, 1993). Additionally, RNA polymerase was upregulated in waterfowl, indicating the high activity of transcription in the fecal microbiota. The predicted functional differences in the microbiota may be driven by the different microbiota profiles induced by different diet compositions among raptors, waders, and waterfowl. Given the influence of pathogens on microbiota composition and microbial interaction patterns, it is urgent to accurately identify the pathogens inhabiting wild birds and to develop new strategies to reduce these pathogens.

In conclusion, we comprehensively examined the fecal microbiota, co-occurrence patterns, and microbiota function in different ecological groups of wild birds using amplicon sequencing and multi-statistical analysis. The differences in microbiota composition, microbiota function, and co-occurrence patterns were associated with diet composition, which may be due to habitat preferences. However, several limitations of this research should be noted: (i) sample collection of wild birds should be increased; (ii) advanced technologies such as metagenomics or metatranscriptomics should be adopted to explore the microbial functional genes, particularly for the pathogens; and (iii) the effect of dietary composition and environment on microbial community structure in wild birds should be quantified. In summary, this research on the fecal bacterial microbiota in raptors, waders, and waterfowl provides valuable insights into the microbial diversity and potential pathogens and provides theoretical knowledge for the protection of endangered animals.

## Data availability statement

The datasets generated for this study can be found in the NCBI/BioProject/PRJNA821628 (https://www.ncbi.nlm.nih.gov/bioproject/PRJNA821628).

## Ethics statement

The Animal Ethics and Welfare Committee (AEWC) of the Wildlife Conservation Center of Baotou and the College of Biological Science and Technology of Baotou Teachers’ College supported the progress of the project.

## Author contributions

CZ and LL designed and performed the experiments. CZ and LG collected the fecal samples. CZ and LB analyzed the data and wrote the manuscript. All authors contributed to the article and approved the final manuscript.

## Footnotes

1
https://github.com/picrust/picrust2
