# Supplementary material for: A comprehensive comparison of fecal microbiota in three ecological bird groups of raptors, waders, and waterfowl
Source: Front Microbiol. 2022 Aug 8;13:919111. doi: 10.3389/fmicb.2022.919111 (PMC9393522; doi:10.3389/fmicb.2022.919111)
Supplement: Supplementary file 1 [file Table_1.DOCX]

Supplementary Material


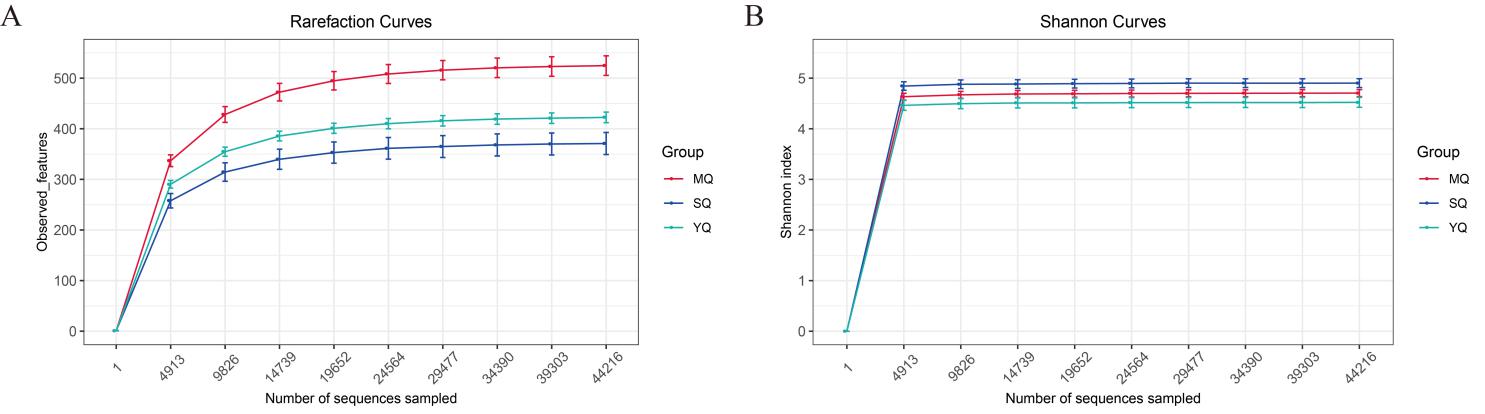


Supplementary Figure 1. The rarefaction (A) and Shannon-Wiener curves (B) for the raptors (MQ), waterfowl (YQ), and waders (SQ).


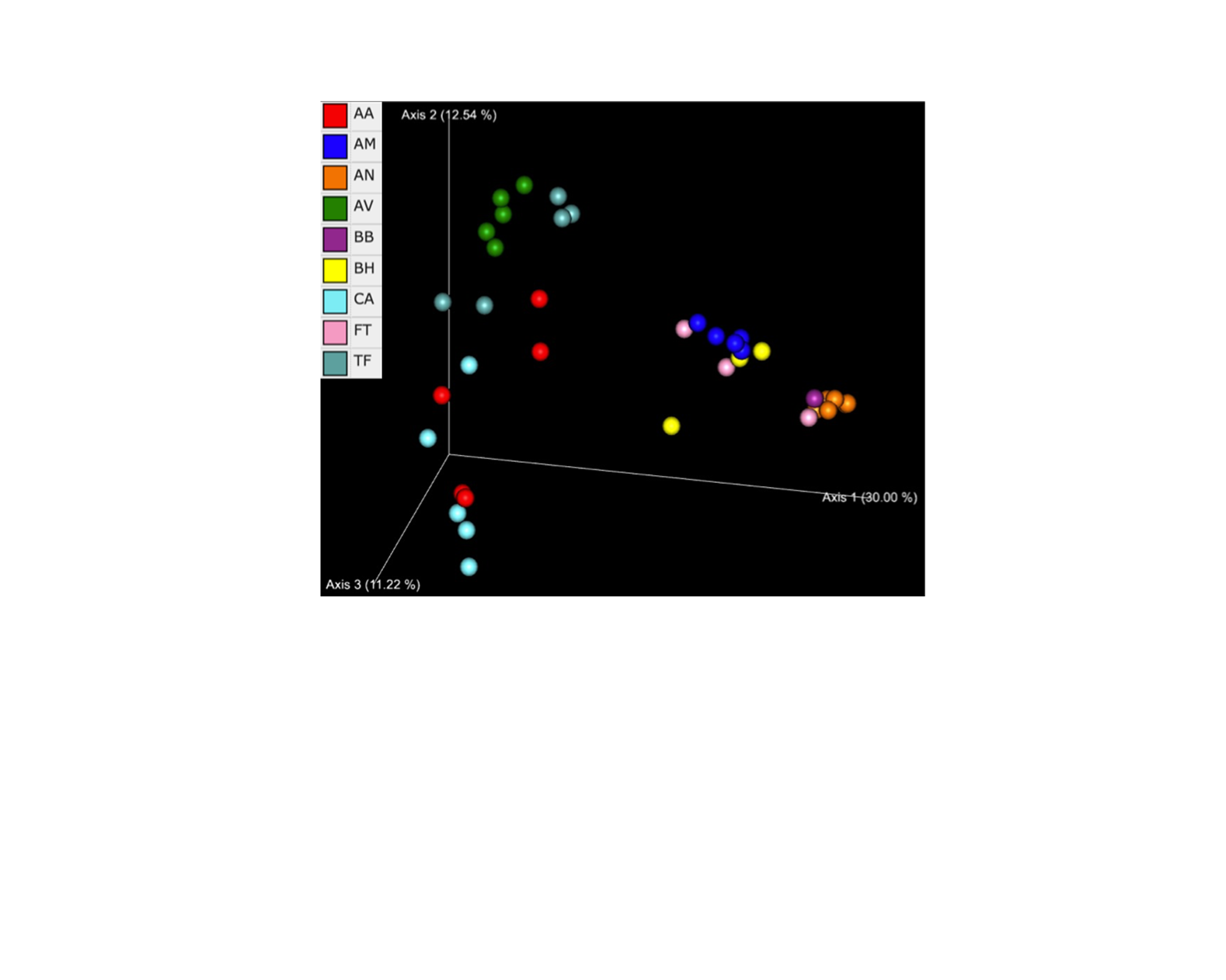


Supplementary Figure 2. Principal coordinate analysis (PCoA) of fecal bacterial communities from 9 bird species.


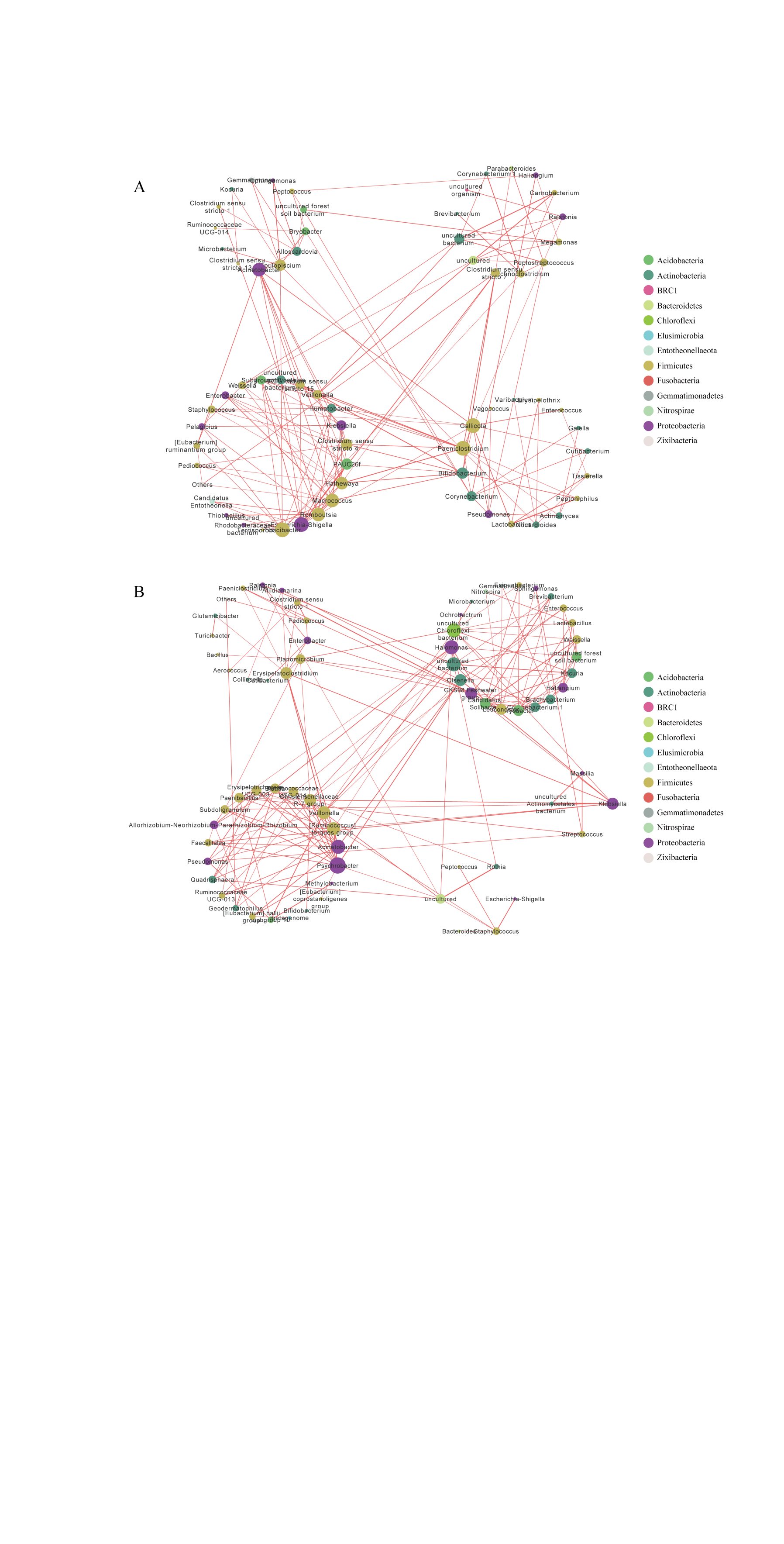


Supplementary Figure 3. Bacterial co-occurrence network in MQ (A) and YQ (B) based on negative correlation analysis at the genus level. Nodes correspond to genus and edges to the correlation. Node size is proportional to the degree number. Node color represents the associated phylum for each genus. Edge width displays the strength of correlation. The red edge indicates a negative correlation. Each large circle represents a module detected by Louvain method.


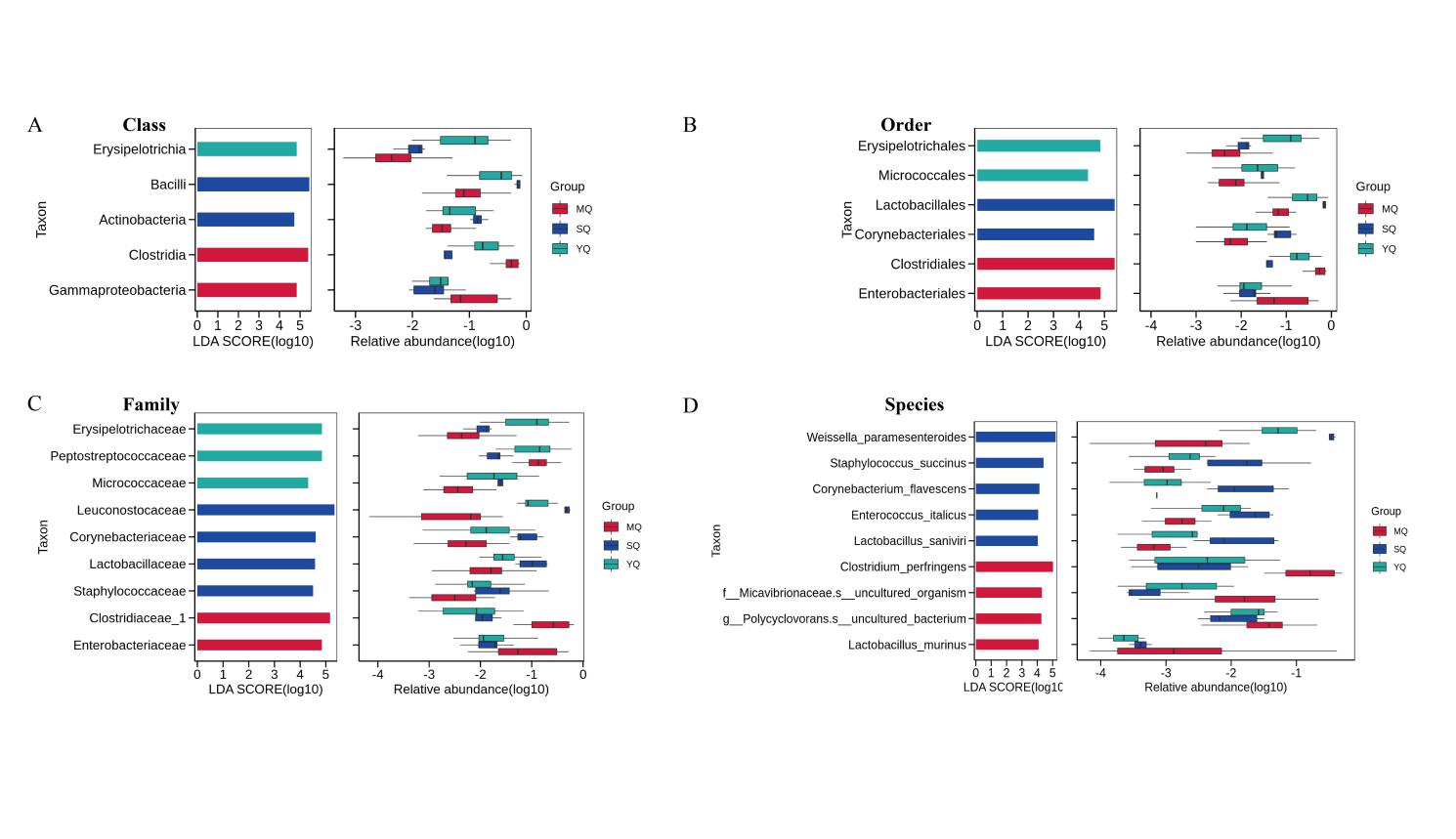


Supplementary Figure 4. The linear discriminant analysis identified significantly different taxon between MQ, SQ and YQ groups at the class (A), order (B), family (C) and species (D) level with a threshold of ﻿LDA score ≥ 4.0 and *p* < 0.05. MQ, raptors; SQ, waders; YQ, waterfowl; LDA: linear discriminant analysis. Left, logarithm score of LDA analysis for each taxon. Right, relative abundance of different taxon.
